# Soft palatal mass containing heterotopic neural tissue causing airway obstruction in a Pierre-Robin sequence patient

**DOI:** 10.1093/jscr/rjab510

**Published:** 2021-12-31

**Authors:** David Z Allen, Lucy X Liu, Kelly Turner, Matthew R Greives, Phuong D Nguyen, Soham Roy

**Affiliations:** Department of Otorhinolaryngology-Head & Neck Surgery, McGovern Medical School, University of Texas Health Science Center at Houston, Houston, TX, USA; Department of Otorhinolaryngology-Head & Neck Surgery, McGovern Medical School, University of Texas Health Science Center at Houston, Houston, TX, USA; Department of Pediatric Plastic Surgery, McGovern Medical School, University of Texas Health Science Center at Houston, Houston, TX, USA; Department of Pediatric Plastic Surgery, McGovern Medical School, University of Texas Health Science Center at Houston, Houston, TX, USA; Department of Pediatric Plastic Surgery, McGovern Medical School, University of Texas Health Science Center at Houston, Houston, TX, USA; Department of Otorhinolaryngology-Head & Neck Surgery, McGovern Medical School, University of Texas Health Science Center at Houston, Houston, TX, USA

**Keywords:** Pierre-Robin sequence, palatal mass, heterotopic neural tissue

## Abstract

Pierre-Robin sequence (PRS) patients frequently exhibit symptoms of airway obstruction due to multiple etiologies, predominantly from glossoptosis and tongue base obstruction. Rarely, these patients can have palatal mass and even rarer is one of neural origin. To date, there are few reports of heterotopic neural tissue causing airway obstruction in literature, and there are only two reports related to PRS. The objective of this report is to detail a PRS patient with obstructive airway symptoms that resolved after removal of a right-sided soft palatal mass containing heterotopic neural tissue. A 5-month-old boy with a past medical history of cleft palate, PRS status-post-mandibular distraction osteogenesis was hospitalized after continuing respiratory distress. Imaging showed a cystic submucosal mass that arose from the right soft palate. Trans-palatal and trans-oral approaches were applied for the removal. The patient tolerated the procedure well and his obstructive events have resolved at follow-up.

## INTRODUCTION

Airway obstruction in a pediatrics patient warrants urgent workup as it can lead to clinical disaster [1, 2]. Specifically, Pierre-Robin sequence (PRS) is a well-known sequence that can lead to airway compromise [2]. While PRS patients predominantly suffer airway obstruction from glossoptosis, and tongue base obstruction, other etiologies should not be ignored. A possible contributor to airway compromise is an oropharyngeal mass, which is rare, however, it necessitates removal. For instance, teratomas in the oropharyngeal cavity are infrequent but can present with obstructive airway concerns and thus must be removed [3]. Heterotopic neural tissue is typically made up of neuroectodermal elements, but its presence in the oropharynx is remarkably rare [4]. Explicitly, the base of the nose is a common place for heterotopic neuroglial tissue to accumulate [5]. However, heterotopic neural tissue leading to airway obstruction in a pediatrics patient is an unusual presentation without much precedent, with only two cases in PRS patients being reported in the literature [4–8].

The present study reports a PRS patient with obstructive airway signs due to a right-sided soft palatal mass containing heterotopic neural tissue, whose symptoms resolved after removal of the mass. The intention of this report is to alert practitioners in the future who come across similar presentations.

## CASE REPORT

A 5-month-old boy, who was born at term with a past medical history of PRS, isolated cleft palate, retrognathia status-post mandibular distraction osteogenesis treatment and gastrostomy tube dependence, presented to the emergency department after worsening apneic events throughout the past few weeks. Of note, he had successfully undergone mandibular distraction and was discharged a month earlier after distractor removal. The patient had a normal direct laryngoscopy and bronchoscopy at that time. After the discharge, the patient was followed by an Otolaryngologist in the clinic who performed routine flexible laryngoscopies and noted a right-sided soft palatal mass.

**Figure 1:**
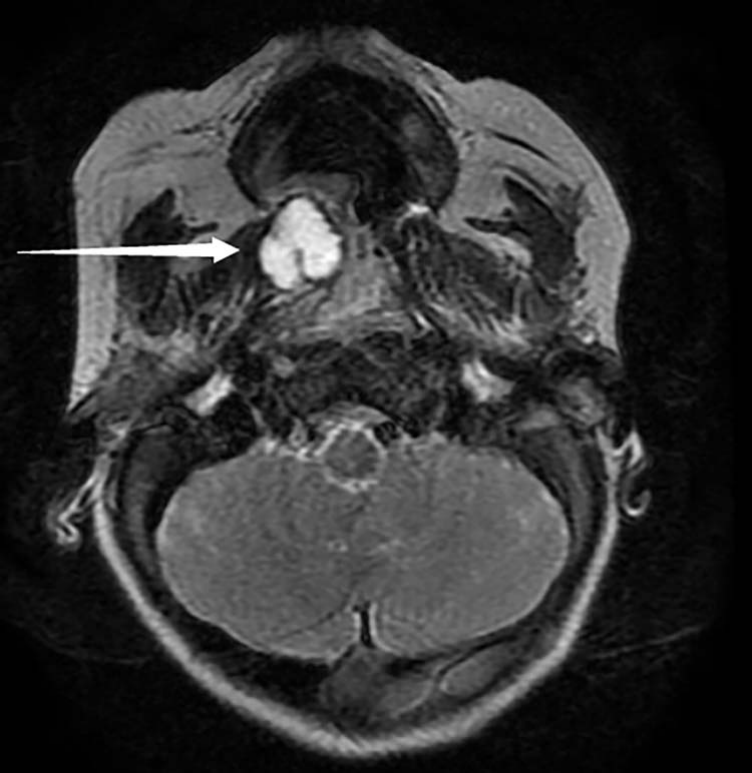
Pictured is an MRI displaying a 1.6 × 1.4 × 1.5-cm cystic submucosal mass arising from the right soft palate.

The patient was hospitalized for respiratory difficulty. During his hospitalization, the patient had episodic desaturations down to 65%, which were improved minimally with suctioning and repositioning. While asleep, the patient developed intermittent increased work of breathing, nasal flaring, subcostal and suprasternal retractions which necessitated increased respiratory support and ICU placement. Inpatient magnetic resonance imaging (MRI) exhibited a 1.6 × 1.4 × 1.5-cm right-sided cystic submucosal mass arising from soft palate with no aggressive features or invasion (Fig. 1).

**Figure 2:**
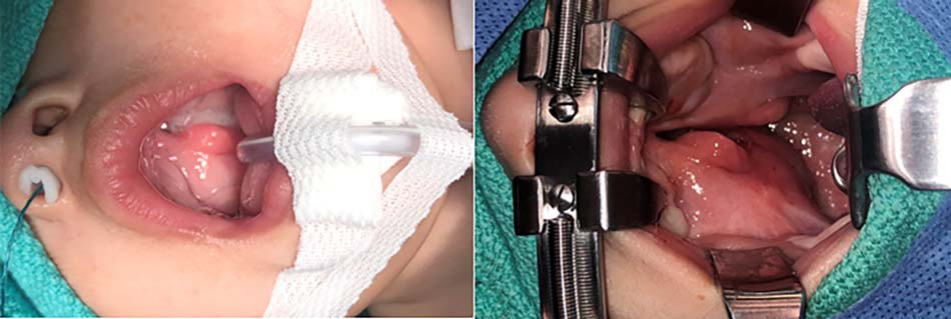
(**a** and **b**) Images of the soft palate mass present prior to the surgical removal; the mass is able to be viewed from the right soft palate that extends into the oropharynx.

Given the persistent obstructive symptoms, surgical removal of the mass was necessitated. During the operation, an obvious cleft palate was visualized along with two uvulas (Fig. 2a and b). The right soft palate had a large and palpable mass arising from the surface that extended inferiorly. Given the extension of the mass inferiorly into the oropharynx, a combined transpalatal and transoral excision was decided. Needle-tip cautery and blunt dissection was performed circumferentially around the mass all the way until the parapharyngeal space (PPS) was encountered along with its associated fat. Intra-operative frozen sections were suspicious for a teratoma and appeared benign while containing neuroglia elements. There was a residual location that was suspicious for a teratoma versus heterotopic neural tissue that was high in the nasopharynx and dangerously close to the lateral pterygopalatine fossae, which had stopped any further resection. Since there was a defect in the lateral aspect of the PPS extending into the nasopharynx, primary closure was performed.

After removal of the mass, a 16-French nasal trumpet was passed down the right nasopharynx, indicating that the nasopharyngeal airway appeared markedly improved. Given the proximity of surgical intervention near the carotid artery and a potential carotid artery rupture, the patient was sent for inpatient observation for 7 days post-operatively. Final surgical pathology of the mass showed benign neuroglial tissue, consistent with heterotopic tissue. While in observation, the patient stabilized to breathing on room air without concern. He was then discharged, and at follow-up, his obstructive events have resolved and his sleeping has dramatically improved.

## DISCUSSION

Airway obstruction in newborn children can be devastating and requires urgent workup. PRS is a well-known clinical sequence that characteristically leads to airway obstruction, which is often managed surgically. Soft palate masses are a rare cause of airway obstruction.

The presented data are scarce regarding oropharyngeal masses containing heterotopic neural tissue, which led to airway obstruction in PRS patients. Some cases of heterotopic neural tissue in the pharyngeal area leading to airway obstruction were reported previously but neither patient had PRS [4]. There are multiple other reports of pediatrics patients with heterotopic neural tissue in the oropharynx, including the soft palate, which led to airway obstruction, however, only two of them had PRS [4, 6].

The soft palatal mass containing heterotopic neural tissue could be lethal. It was reported a PRS newborn with a huge left-sided soft palatal mass extending into the infratemporal fossa passed away due to a tracheostomy dislodgement [6]. The mass was only debulked instead of a complete removal, probably due to the large size. The case highlighted the necessity of the surgical removal of soft palatal mass before it is too late.

Another report detailed a case of heterotopic neural tissue involving the PPS in a PRS patient which led to airway obstruction, which had also been successfully removed [7]. The report differs from ours as that patient was a newborn (<2 months old) and had not undergone any form of treatment for PRS yet. Additionally, the mass did not arise from the soft palate and instead just abutted the soft palate and the cleft was partial [7].

The pathogenesis of heterotopic neuroglial tissue is unclear, but several mechanisms have been proposed. One hypothesis is that during early embryogenesis, displacement of totipotent neuroectodermal cells arises and develops into mature neural tissue. Another hypothesis is abnormal migration of glial cells from the olfactory bulbs [4].

The present report has described an unusual source of airway obstruction in a PRS patient who underwent corrective airway surgery. We encourage clinicians to explore other etiologies in PRS patients with continuing symptoms of airway obstruction.

Heterotopic neural tissue resembling a mass arising from the soft palate can lead to respiratory compromise in PRS patients. Close follow-up and surgical intervention are warranted for these patients with necessary surgical preparation and planning.
